# Optimization of Heavy Chain and Light Chain Signal Peptides for High Level Expression of Therapeutic Antibodies in CHO Cells

**DOI:** 10.1371/journal.pone.0116878

**Published:** 2015-02-23

**Authors:** Ryan Haryadi, Steven Ho, Yee Jiun Kok, Helen X. Pu, Lu Zheng, Natasha A. Pereira, Bin Li, Xuezhi Bi, Lin-Tang Goh, Yuansheng Yang, Zhiwei Song

**Affiliations:** Bioprocessing Technology Institute, Agency for Science, Technology and Research (A*STAR), Singapore, Singapore; Naval Research Laboratory, UNITED STATES

## Abstract

Translocation of a nascent protein from the cytosol into the ER mediated by its signal peptide is a critical step in protein secretion. The aim of this work was to develop a platform technology to optimize the signal peptides for high level production of therapeutic antibodies in CHO cells. A database of signal peptides from a large number of human immunoglobulin (Ig) heavy chain (HC) and kappa light chain (LC) was generated. Most of the HC signal peptides contain 19 amino acids which can be divided into three domains and the LC signal peptides contain 22 amino acids. The signal peptides were then clustered according to sequence similarity. Based on the clustering, 8 HC and 2 LC signal peptides were analyzed for their impacts on the production of 5-top selling antibody therapeutics, namely, Herceptin, Avastin, Remicade, Rituxan, and Humira. The best HC and LC signal peptides for producing these 5 antibodies were identified. The optimized signal peptides for Rituxan is 2-fold better compared to its native signal peptides which are available in the public database. Substitution of a single amino acid in the optimized HC signal peptide for Avastin reduced its production significantly. Mass spectrometry analyses revealed that all optimized signal peptides are accurately removed in the mature antibodies. The results presented in this report are particularly important for the production of these 5 antibodies as biosimilar drugs. They also have the potential to be the best signal peptides for the production of new antibodies in CHO cells.

## Introduction

Recombinant monoclonal antibodies produced by CHO cells represent the most rapidly growing class of biotherapeutics. The annual sales of the *top-selling antibody drugs*, such as Humira, Remicade, Avastin, Rituxan and Herceptin, are over or close to $ 10 billion now while many potential blockbusters are being investigated in clinical trials [1, 2]. As a consequence, the demand for the production of large quantities of recombinant antibody therapeutics has increased dramatically and extensive efforts have been directed at improving their yields in mammalian cells [3].

Antibodies, like many other proteins, are secreted from the cells via the co-translational translocation pathway. In eukaryotes, a signal peptide that contains 5–30 amino acids present at the N-terminus of nascent proteins is recognized by the signal recognition particle (SRP) in the cytosol while the protein is still being synthesized on the ribosome. The SRP then delivers the SRP-ribosome-nascent chain (SRP-RNC) complex to the SRP-receptor (SR) in the endoplasmic reticulum (ER) membrane. GTP-dependent mechanisms then deliver the RNC complex to a membrane-bound translocon which allows translocation of the growing polypeptide chain into the lumen of the ER. After crossing the ER membrane, the signal peptide is cleaved off by a signal peptide peptidase (SPP) for reviews see [4–10].

The translocation of secretory proteins into the lumen of the ER has been identified as a bottleneck within the secretory pathway and thus represents a key issue that needs to be resolved to achieve robust production of recombinant proteins. Studies have shown that signal peptides are extremely heterogeneous and many signal peptides are functionally interchangeable even between different species[11, 12]. On the other hand, different signal peptides can exert profoundly different impact on protein secretion [13]. Thus the efficiency of protein secretion can be strongly affected by the signal peptide [12–17]. These observations are highly indicative of the importance of signal peptide optimization when aiming to produce maximal amounts of recombinant proteins in a mammalian system.

Human IgG antibodies consist of two identical heavy chains (HCs) and two identical light chains (LCs). Efficient expression of the HC and LC requires appropriate signal peptides for the transport of the HC and the LC polypeptides into the ER for proper folding, assembly and post-translational modification. However, sequence information of most recombinant therapeutic antibodies in publicly available databases rarely includes the signal peptides. Even in cases where they are available, the native signal peptide may not be optimal and thus may require further improvement. Furthermore, single-chain Fv antibodies that are frequently isolated from platforms such as phage display libraries do not include signal peptides. In addition, signal peptides could be host-cell specific. Due to the limited signal peptide information available for the production of monoclonal antibodies, the aim of this work was to develop a platform to optimize signal peptides for the production of recombinant antibodies in CHO cells. Five top-selling therapeutic antibodies which are also popular biosimilar candidates for many biotech companies, namely Herceptin (trastuzumab), Avastin (bevacizumab), Remicade (infliximab), Rituxan (rituximab) and Humira (adalimumab), were used as model molecules in this study. We generated a signal peptide database of human antibody heavy chains and light chains. Using a bioinformatics approach, the signal peptides were then clustered based on sequence similarity. Eight HC signal peptides and two LC signal peptides were selected for the assessment of their impacts on antibody production. The optimized signal peptides for Herceptin, Avastin, Remicade, Rituxan and Humira were identified. This platform technology may also be used for the production of other recombinant antibodies in CHO cells.

## Materials and Methods

### Generation of HC and LC Constructs for Expressing Herceptin, Avastin, Remicade, Rituxan and Humira

The variable regions of the HC and LC of the 5 therapeutic antibodies were determined based on publicly available information. The DNA fragments encoding these variable regions were fused to the constant regions of human IgG1 HC or kappa LC by overlap PCR. Signal peptides from 172 human antibody HCs were analyzed based on sequence similarity. These antibodies include human IgG, IgM, IgD, IgA and IgE. Signal peptides from 62 human kappa chains were also analyzed. Only the HCs and LCs with complete cDNA sequences from the PubMed database were used in this study. The phylogenetic trees of the HC and LC signal peptides are shown in S1 Fig. Detailed sequence information of all the signal peptides shown in S1 Fig. is shown in S1 Table. Based on these results, eight HC signal peptides (H1 to H8) and two kappa LC signal peptides (L1 and L2) were chosen and compared for their impact on antibody secretion (Table 1). Each antibody HC and LC was fused to eight HC signal peptides and two LC signal peptides respectively by overlap PCR and cloned into pcDNA3.1 (Life Technologies, Inc.) or an EF1-α promoter-based expression vector.

**Table 1:** The amino acid and DNA sequences for the heavy and light chain signal peptides selected in this study.

**Heavy chain**	**Amino acid sequence**	**DNA sequence**
**H1**	MELGLSWIFLLAILKGVQC	ATGGAGTTGGGACTGAGCTGGATTTTCCTTTTGGCTATTTTAAAAGGTGTCCAGTGT
**H2**	MELGLRWVFLVAILEGVQC	ATGGAACTGGGGCTCCGCTGGGTTTTCCTTGTTGCTATTTTAGAAGGTGTCCAGTGT
**H3**	MKHLWFFLLLVAAPRWVLS	ATGAAACACCTGTGGTTCTTCCTCCTGCTGGTGGCAGCTCCCAGATGGGTCCTGTCC
**H4**	MDWTWRILFLVAAATGAHS	ATGGACTGGACCTGGAGGATCCTCTTCTTGGTGGCAGCAGCAACAGGTGCCCACTCG
**H5**	MDWTWRFLFVVAAATGVQS	ATGGACTGGACCTGGAGGTTCCTCTTTGTGGTGGCAGCAGCTACAGGTGTCCAGTCC
**H6**	MEFGLSWLFLVAILKGVQC	ATGGAGTTTGGGCTGAGCTGGCTTTTTCTTGTGGCGATTCTAAAAGGTGTCCAGTGT
**H7**	MEFGLSWVFLVALFRGVQC	ATGGAGTTTGGGCTGAGCTGGGTTTTCCTCGTTGCTCTTTTTAGAGGTGTCCAGTGT
**H8**	MDLLHKNMKHLWFFLLLVAAPRWVLS	ATGGACCTCCTGCACAAGAACATGAAACACCTGTGGTTCTTCCTCCTCCTGGTGGCAGCTCCCAGATGGGTGCTGTCC

**Light chain**	**Amino acid sequence**	**DNA sequence**
**L1**	MDMRVPAQLLGLLLLWLSGARC	ATGGACATGAGGGTCCCTGCTCAGCTCCTGGGGCTCCTGCTGCTCTGGCTCTCAGGTGCCAGATGT
**L2**	MKYLLPTAAAGLLLLAAQPAMA	ATGAAATACCTATTGCCTACGGCAGCCGCTGGATTGTTATTACTCGCGGCCCAGCCGGCCATGGCC

### Transient Expression of the 5 Antibodies with Different Signal Peptides in CHO-K1 Cells

For transient expression of the 5 antibodies with different signal peptides, 6×10^5^ CHO-K1 cells were seeded in 6-well plates 24 h prior to transfection. Duplicate transfections for each pair of heavy and light chain vectors were performed using FuGENE 6 reagent (Roche Applied Science, Rotkreuz, Switzerland). FuGENE 6 reagent to plasmid ratio was 6 μl:2 μg. 1 μg of each HC and LC bearing plasmids was used in each transfection. To normalize transfection efficiency, a third transfection was carried out in parallel for each heavy and light chain pair with an additional 0.2 μg of plasmid bearing a gene coding for green fluorescence protein (GFP), pmaxGFP (Lonza, Cologne, Germany). The transient expression experiments described above were repeated once to ensure that the results were reliable.

### Quantification of Secreted Antibody by ELISA

At 48 h post-transfection, supernatants from cultures transfected with only heavy and light chain vectors (without pmaxGFP) were collected for analysis of mAb concentration using enzyme-linked immunosorbent assay (ELISA) in 96-well plate (Nunc) as described previously [18, 19]. Briefly, the plate was first coated with capture antibody which is affinity-purified goat anti-human IgA + IgG + IgM (H + L) antibody (KPL, Gaithersburg, MD) in PBS at 37°C for 1 h. Following three washes with PBS containing 1% bovine serum albumin (BSA) (Sigma), 300 μL of blocking buffer (3% BSA in PBS) were added to each well and incubated at 37°C for 1 h. The plate was then washed again as described above. Fifty microliters of human antibody standard (IgG1, kappa) (Sigma) and diluted samples were added in duplicates and incubated at 37°C for 1 h. After three washes, 50 μL of goat anti-human IgG (Fc specific) conjugated to alkaline phosphatase (Sigma) was added and incubated at 37°C for 1 h. After washing, 50 μL of p-nitrophenyl phosphate substrate (Sigma) were added and incubated at room temperature for 30 min. The reaction was stopped by 1 M NaOH and absorbance at 405 nm (reference 630 nm) was measured on a Universal Microplate Spectrophotometer (Bio-TEK1 Instruments, Winooski, VT). Cells from cultures co-transfected with heavy and light chain vectors and the pmaxGFP vector were collected to measure the fluorescence intensity using a FACS Calibur (Becton Dickinson, Bedford, MA) [19]. The ELISA results were normalized to the mean fluorescence intensity of GFP as described by Ho et al. [19].

### Generation of Avastin HC Constructs with Variants of Signal Peptide H7

Six Avastin HC constructs with altered signal peptides that have one or more amino acid residues mutated from the optimized H7, designated H7a—H7f, were generated. The mutants were engineered using QuikChange II XL site-directed mutagenesis kit (Agilent, Santa Clara, CA) and corresponding primer pairs. The impact of these H7 variants on Avastin production was determined by transient transfection into CHO cells followed by ELISA as described earlier.

### Production and Purification of Recombinant Antibodies with Optimized Signal Peptides for Mass Spectrometry Analyses

CHO-K1 cells were cultured in 15 cm dishes until almost confluence and transfected with each pair of the optimized heavy and light chain constructs using lipofectamine 2000 reagent (Life Technologies). Forty-eight hours after transfection, cells were selected in medium supplemented with 800 μg/ml of G418 (Life Technologies). After 14 days of selection, surviving cells were pooled and adapted to suspension culture by gradually reducing the concentration of fetal bovine serum from 10% to 5% to 2.5% to 1% in the suspension culture medium comprising 50% (v/v) of CD CHO (Life Technologies) and 50% (v/v) of HyClone PF-CHO MPS (Thermo Scientific, Asheville, NC), supplemented with 6 mM L-glutamine (Life Technologies) and 0.1% Pluronic F-68 (Life Technologies), as well as 400 μg / ml of G418. These cells which are referred to as the “stably transfected pools” were then seeded at 2.5x10^5^ cells/ml in 200 ml of suspension culture medium in 1 L shake flasks. After 6-days of culturing, the medium was collected and the antibodies were purified with an *FPLC AKTA Purifier* (GE Healthcare, Pittsburgh, PA) on a HiTrap Protein A HP column (GE Healthcare) which was equilibrated with 20 mM, pH 7.0 sodium phosphate buffer. The antibody was eluted with 0.1 M, pH2.7 glycine buffer.

### NanoLC-MS/MS to Analyze the Cleavage of the Signal Peptide in Secreted Antibodies

Diafiltration cartridges (30 kDa) (Millipore, Billerica, MA) were used to concentrate 20 μg of each antibody produced by the stably transfected pools. Antibodies were then supplemented with 20 mM triethylammonium bicarbonate, pH 8.5, reduced with 30 mM tris(2-carboxyethyl)phosphine (TCEP) at 60°C for 1 h, and alkylated with 60 mM iodoacetamide at room temperature in the dark for 40 min. Digestion was carried out using sequencing-grade modified trypsin (1:25) (Promega, Madison, WI) overnight at 37°C. Peptide samples were dried down in Savant SpeedVac (Thermo Scientific), and resuspended with 25 μl buffer A (0.1% formic acid).

Nanoscale liquid chromatography (NanoLC) was performed on nanoACQUITY UPLC System (Waters, Milford, MA). Peptide sample (2 μl) was loaded onto Symmetry C18 trapping column, 5 μm, 180 μm x 20mm (Waters) and desalted for 8 min with 2% buffer B (0.1% formic acid in acetonitrile) at 8 μl/min. Trapping column was subsequently switched online to nanoACQUITY UPLC BEH130 C18 column, 1.7 μm, 75 μm x 150 mm (Waters), and peptides were separated at 300 nl/min with a gradient consisting of 60 min 2–28% buffer B, 8 min 28–40% buffer B and 5 min 97% buffer B.

Mass spectrometer (MS) detection was performed on a LTQ Orbitrap Velos MS (Thermo Scientific) operating in CID top 10 mode, with nanoelectrospray potential at 1.7 kV. Full scan MS spectra (from m/z 300–1,800) were obtained by data dependent acquisition with resolution set at 60,000. The 10 most intense peptide ions with charge state ≥2 were sequentially fragmented with normalized collision energy of 35 V. Minimum signal threshold for MS/MS was set at 500 counts, activation q value at 0.25 and activation time at 10 ms. Ion trap and orbitrap maximal injection times were set to 100 ms and 10 ms respectively.

Raw data files were processed by Proteome Discoverer (v1.3.0.339, Thermo Scientific) using SEQUEST algorithm, and searched against respective compiled databases consisting of sequentially shortened antibody sequences from the N-terminal. N-terminal peptide quantifications were obtained using Xcalibur (v2.2, Thermo Scientific) by calculating peak area of extracted ion chromatogram (XIC) with mass tolerance of 10 ppm.

## Results

### Evaluation of Human Immunoglobulin Signal Peptides for Antibody Secretion in CHO-K1 cells

Sequences of human Ig HC and kappa LC cDNAs with complete coding regions were collected from the PubMed database. In total, 172 Ig HCs and 62 kappa LCs were gathered. Majority of the HC signal peptides contain 19 amino acids and all of the kappa LC signal peptides contain 22 amino acids. A database of signal peptide sequences was generated using these HCs and LCs. The signal peptides were then aligned based on sequence similarity using BioEdit (http://www.mbio.ncsu.edu/bioedit/bioedit.html) and the phylogenetic trees of the HC and LC signal peptides are shown in S1 Fig. Detailed information on all these signal peptides is listed in S1 Table. Based on this analysis, eight HC signal peptides (H1–H8) and two kappa LC signal peptides (L1 and L2) were selected. The amino acid sequences and the corresponding DNA sequences of these signal peptides are shown in Table 1. The signal peptides were then assessed for their impact on antibody secretion in CHO-K1 cells. The variable regions and the constant regions of the HC and LC of Herceptin, Avastin, Remicade, Rituxan and Humira were generated based on publicly available information. Each antibody HC was then fused to eight signal peptides (H1–H8) to generate eight different HC constructs. Each antibody LC was fused to two signal peptides (L1 and L2) to generate two LC constructs.

To analyze the impact of signal peptides on the secretion of each antibody, 16 heavy and light chain combinations for each antibody were transfected into CHO-K1 cells. Duplicate transfections for each pair of heavy and light chain combination were performed. To normalize transfection efficiency, a third transfection was also performed. In this transfection, in addition to the heavy and light chain constructs, a construct expressing GFP was also included in the transfection as the control for transfection efficiency as described previously [18, 19].

**Fig 1:**
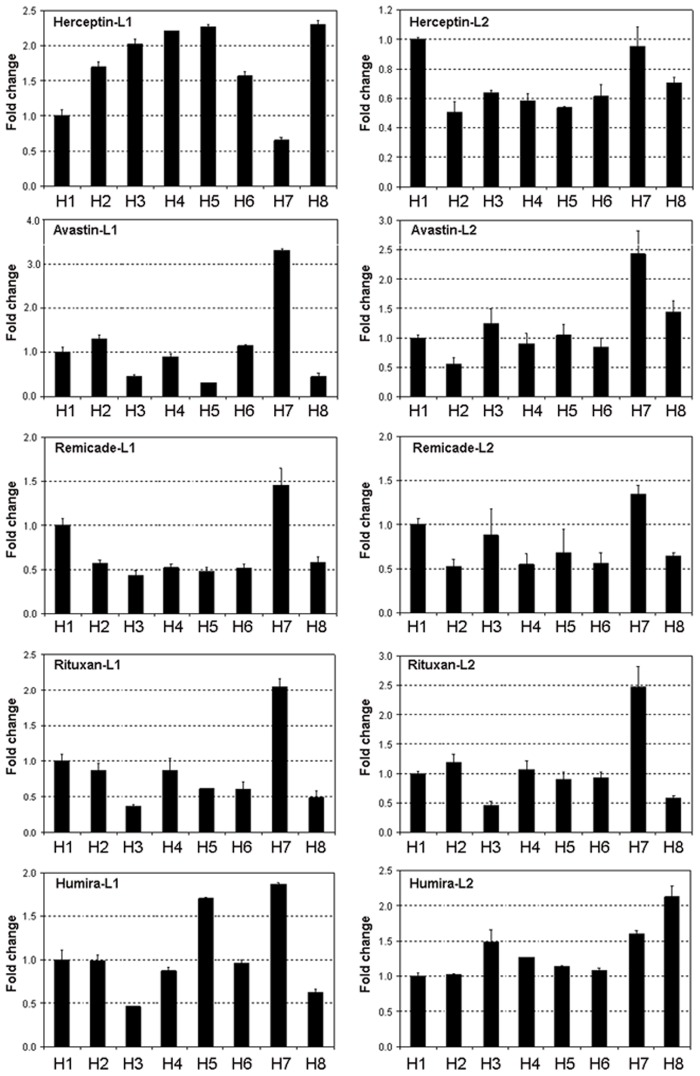
Relative amounts of the antibodies produced after normalization with GFP. To minimize the impact of transfection efficiency on antibody production, data presented in Fig. 1 were normalized with GFP levels for each transfection pair. For each antibody, 8 HC constructs were paired with L1 LC (left panel) or L2 LC (right panel), respectively. All values were expressed as fold-change relative to H1 which was set as 1.

The antibody concentration in each conditioned medium was determined 2 days after transfection by ELISA. The raw data of the ELISA results and the expression levels of GFP are shown in S2 Fig. Within each box, the secretion efficiency of the antibody with signal peptide H1 to H8 was compared. For each heavy and light chain transfection pair, the mean fluorescence intensity of GFP was measured as a control for transfection efficiency. The fold changes in the concentrations of each antibody detected in the medium is normalized to the mean fluorescence intensity of GFP and then compared to that of H1 (Fig. 1). The error bars are the standard deviation of measurements from four independent transfections. These results eliminated the differences in antibody expression caused by varying transfection efficiencies and allowed us to draw direct comparisons between 8 heavy chain signal peptides. As shown clearly, the amounts of the antibody produced are highly dependent on the signal peptide used. Interestingly, the HC signal peptide 7 (H7) resulted in significantly increased secretion for Avastin, Remicade, Rituxan and Humira. This observation was seen with the use of both LC signal peptides L1 and L2.

**Fig 2:**
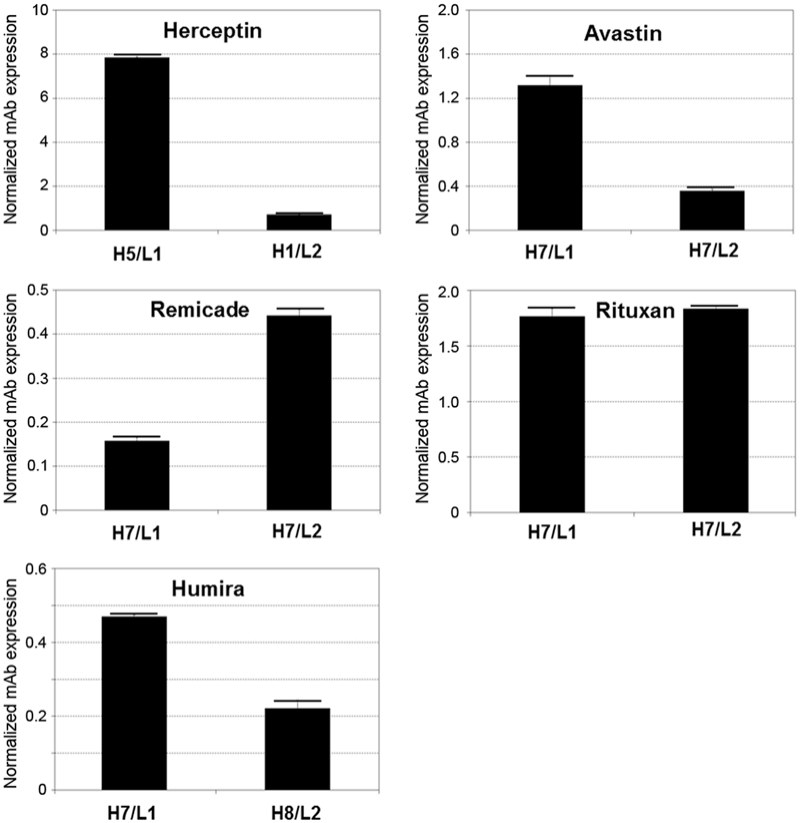
Comparisons between the highest titers produced by L1 and L2 LCs for each antibody obtained using IgG ELISA. The average amounts of the antibody produced were normalized to the GFP levels in the control experiments.

To determine the best signal peptide pair for each antibody, the HC signal peptide that produced the highest amount of antibody when paired with L1 LC was compared with the HC that produced the highest amount of antibody when paired with L2 LC. The selected HC and LC constructs were transfected again into CHO-K1 cells and the antibodies produced were determined by ELISA as described earlier. The results shown in Fig. 2 confirmed that the best signal peptide combination for the production of Herceptin, Avastin, Remicade, Rituxan and Humira are H5/L1, H7/L1, H7/L2, H7/L2 and H7/L1 respectively. Results shown in Fig. 2 also suggest that under these experimental conditions with our optimized signal peptides, Herceptin is produced most efficiently in CHO cells, followed by Rituxan, Avastin, Humira, and lastly Remicade. The same order with respect to productivity was confirmed when stably transfected pools were used to produce these antibodies (data not shown).

### The Optimized Signal Peptides for Rituxan Perform Better than Its Native Signal Peptides

**Fig 3:**
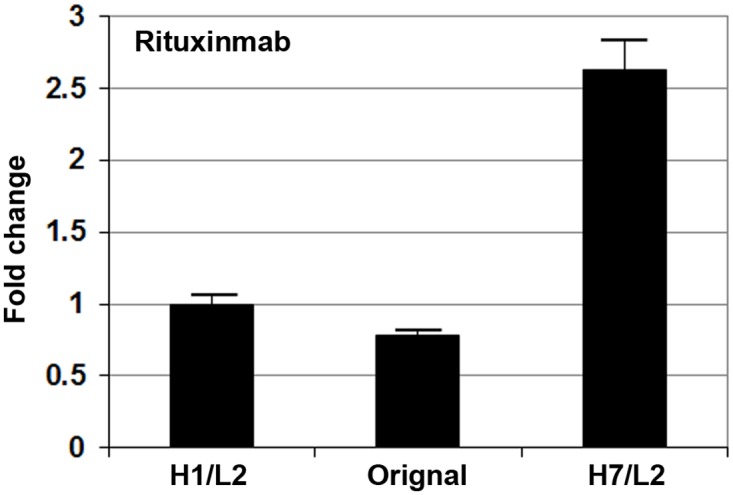
A comparison between the native and the optimized signal peptides on their impact on production of Rituxan. The amounts of the Rituxan antibody in the conditioned media were determined by ELISA. The H1/L2 pair was included in the experiment as a control to ensure the reproducibility of the experiment.

Among the five antibodies that were studied in this work, Rituxan is the only antibody for which the native heavy and light chain signal peptide information is available in public database, the sequences of which are MGWSLILLFLVAVATRVLS and MDFQVQIISFLLISASVIMSRG, respectively. It is crucial to determine whether our optimized signal peptides are capable of enhancing secretion of Rituxan compared to its native signal peptides. Therefore, a comparison between the original signal peptides and our optimized signal peptides (H7/L2) on the production of Rituxan was performed by a transient transfection experiment. Because many factors (such as plasmid quality, transfection efficiency, error in ELISA, etc.) can affect the amount of antibody found in conditioned medium, it is important to include effective controls in order to ensure data consistency in different experiments. Therefore, Rituxan-producing constructs H1/L2 were also included in this comparison experiment as a control. If the ratio of the Rituxan produced by H7/L2 to that by H1/L2 is also about 2.5 as shown earlier in Fig. 1, the results of the experiment is considered consistent. Indeed, as shown in Fig. 3, the H7/L2 to H1/L2 ratio is about 2.5. The ELISA results also show that our optimized signal peptides (H7/L2) resulted in more than a 2-fold increase in antibody titre compared to the original signal peptides.

### Sequence Comparisons of the Eight Ig HC Signal Peptides

**Fig 4:**
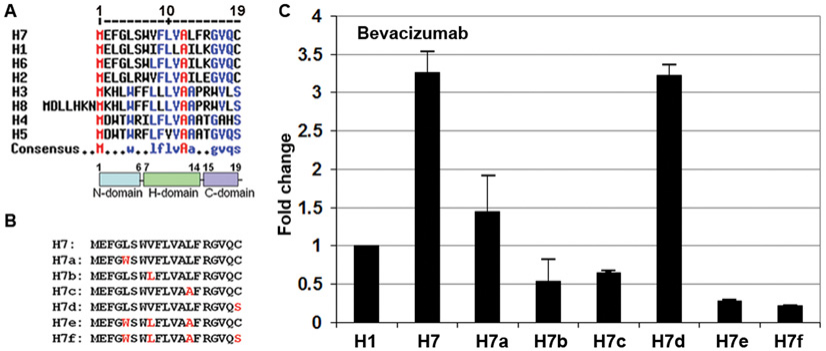
Analysis of the optimized signal peptide H7 using Avastin as a model molecule. (**A**) A sequence comparison of the HC signal peptides with H7 revealed several highly conserved amino acid residues: M…W.. LFLVAA…GVQS. Except for H8, all other signal peptides contain 19 amino acids which can be divided into N-domain, H-domain and C-domain. (**B**) Six H7 variants (H7a to H7f) were generated. Mutated amino acids are highlighted in red. (**C**) The amounts of the Avastin antibody detected in the conditioned media when different HC constructs were used as determined by ELISA. H1 was included in the experiment as a control to ensure the reproducibility of the experiment.

It has been suggested that the signal peptides contain 3 domains, the positively charged N-terminal domain (N-domain), followed by the hydrophobic domain (H-domain) and the polar C-terminal domain (C-domain) [20, 21]. The sequence alignment analysis of the eight HC signal peptides identified in this study is shown in Fig. 4A. All these signal peptides contain 19 amino acids except for H8 which contains 26 amino acids. In all the signal peptides except for H3, the second amino acid is a negatively charged glutamic acid or aspartic acid (E or D), however, it is a lysine (K) at the same position in H3. In fact, in almost all of 172 Ig HCs in our database, the second amino acid is either E or D. The second amino acid is a K in almost all signal peptides in cluster 3 represented by H3. Amino acid 7 to 14 forms the hydrophobic H-domain and amino acid 15 to 19 forms the C-domain. Four signal peptides (H1, H2, H6, H7) terminate with a cysteine (C) residue, whereas the other four terminate with a serine (S) residue. There is only one negatively charged amino acid (E), and no positively charged amino acid, in the N-domains of the signal peptide clusters 1, 6 and 7 (represented by H1, H6 and H7). Therefore, not all N-domains of the signal peptides are positively charged.

### Characterization of the Optimal Signal Peptide for Avastin (H7)

The sequence alignment of 8 HC signal peptides selected in this study (Fig. 4A) revealed several highly conserved amino acids: **M…w/L..lflvAa..gvqs/c**. Three amino acid residues in H7 were divergent from the highly conserved sequence (**MEFGLSWVFLVALFRGVQC**), namely L5, V8 and L13. To investigate the functional significance of these amino acids, six H7 variants (H7a—H7f) were created (Fig. 4B). H7a carries a L5W mutation, because W is another highly conserved amino acid at position 5 (Fig. 4B). Similarly, H7b carries a V8L mutation and H7c carries a L13A mutation. To compare the difference between C and S at the cleavage site, H7d which carries a C19S mutation was generated. In H7e, three amino acids in H7 were mutated and in H7f, all four amino acids were mutated. These mutated signal peptides were fused to the Avastin HCs and co-transfected with Avastin L1 LC into CHO-K1 cells and the antibody produced in each transfection was determined by ELISA. For the same reason stated earlier (Fig. 3), Avastin heavy chain with H1 signal peptide was also included in this experiment as a control. Interestingly, the results show that the substitution of any of the three amino acids (H7a, H7b and H7c) dramatically reduced the production of the antibody (Fig. 4C), suggesting that each of these three amino acids in H7 is important for its function as a signal peptide for Avastin. Substituting all three amino acids together (H7e and H7f) further reduced the secretion of Avastin. In contrast, substitution of cysteine to serine (H7d) at the cleavage site does not affect the secretion of the antibody.

### Analysis of Antibody Heterogeneity due to Cleavage of the Signal Peptides

In addition to secretion efficiency, we also attempted to address the industrial problem of cleavage heterogeneity which occurs as a result of non-specific cleavage of the signal peptide by SPP. This phenomenon can lead to either elongation or truncation of the N-terminus of the heavy and light chains which may not be suitable for biopharmaceutical therapeutics [22, 23]. Each antibody with its optimized heavy and light chain signal peptides was produced by stably transfected pools of CHO-K1 cells and purified by protein A affinity chromatography. Purified antibodies were subsequently digested by trypsin, and the resultant peptides were analysed by mass spectrometry.

**Fig 5:**
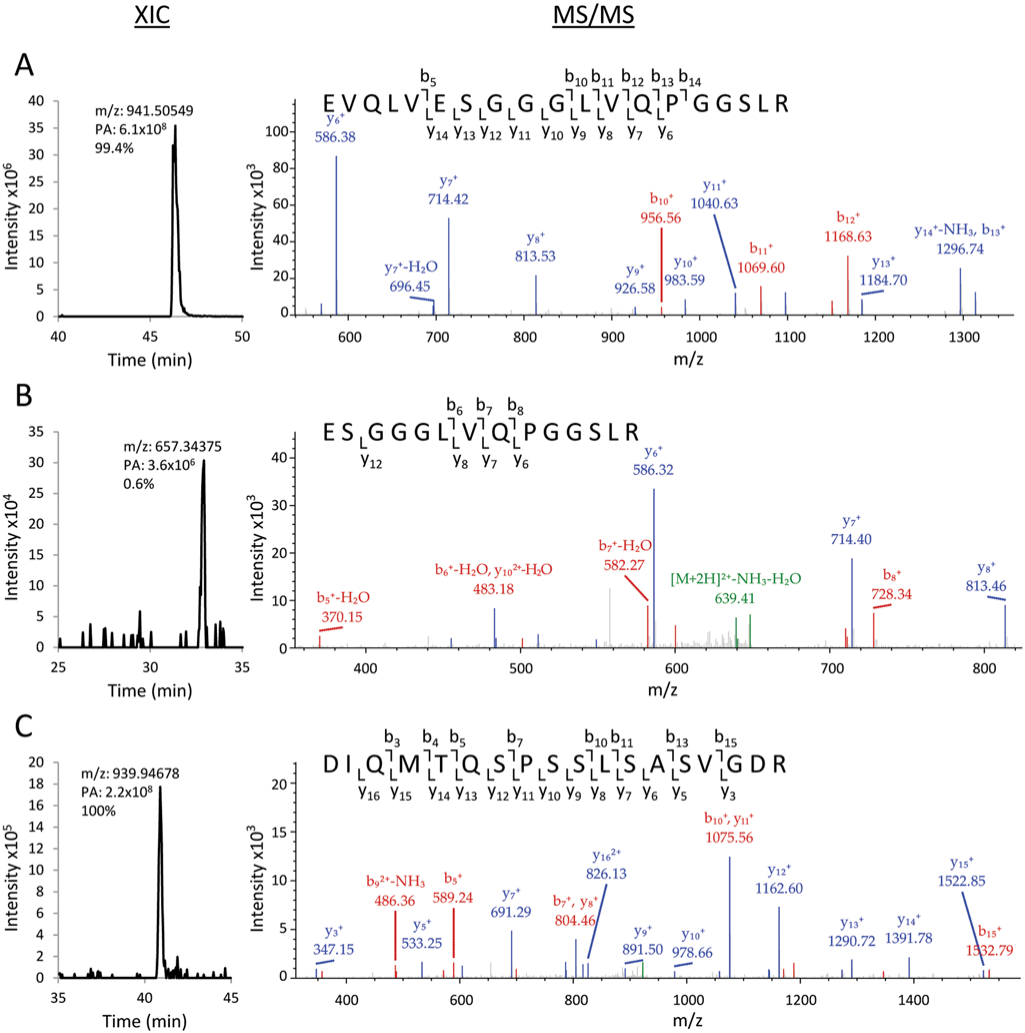
MS analysis of Avastin N-terminal peptides. Extracted ion chromatogram (XIC) peaks and MS/MS fragmentation spectra matching to b- and y-ions series of (**A**) HC N-terminal peptide, (**B**) erroneously processed HC N-terminal peptide resulting from an alternative cleavage site 5 residues downstream of expected cleavage site, and (**C**) LC N-terminal peptide. Mass/charge ratio (m/z) of each N-terminal peptide, peak area (PA) and its percentage representation relative to total N-terminal peptides detected for corresponding heavy or light chain polypeptides are shown in the XIC spectra.

Detection of alternative cleavage sites of the N-terminal peptides of both heavy and light chains of each antibody were carried out by tryptic peptide mapping using LC-MS/MS. N-terminal peptides were identified by high-resolution tandem mass spectrometry (MS/MS), and corresponding peptide precursor peak areas from extracted ion chromatograms (XICs) were used for relative quantification [24]. The results obtained for Avastin showed that the correct HC N-terminal peptide EVQLVESGGGLVQPGGSLR (*m/z* 941.51) accounted for 99.4% of total HC N-terminal peptides detected, while an erroneously processed peptide ESGGGLVQPGGSLR (*m/z* 657.35), cleaved 5 residues downstream of the expected cleavage site, accounted for 0.6% (Fig. 5A & 5B). For the LC, only the correctly processed N-terminal peptide DIQMTQSPSSLSASVGDR (*m/z* of 939.95) was detected (Fig. 5C), thus suggesting absence of alternative cleavage site in signal peptide processing. N-terminal peptides from other antibodies were similarly identified and quantified in triplicate mass spectrometry analyses. The results are summarized in Table 2 and the detailed breakdown is shown in S2 Table. As depicted, the optimized signal sequences used in the expression of the antibodies did not give rise to significant cleavage heterogeneity of the signal peptides. Efficacy of N-terminal processing at the expected cleavage site ranged from ~99.2% to 100%, while N-terminal peptides resulting from erroneous cleavage, when present and summed, accounted for less than 1% of total N-terminal peptide population.

**Table 2:** Proportion of N-terminal peptide(s) quantified by triplicate analyses using mass spectrometry.

Antibody	Signal peptide	Sequence (N-terminal)	Average % [SEM]
Avastin—HC	H7	…QC ^▼^EVQLVESGGGLVQPGGSLR	99.38 [*4.5e-4*]
		…QCEVQLV^▽^ESGGGLVQPGGSLR	0.62 [*4.5e-4*]
Avastin—LC	L1	…RC ^▼^DIQMTQSPSSLSASVGDR	100 [*nil*]
Herceptin—HC	H5	…QS ^▼^EVQLVESGGGLVQPGGSLR	99.91 [*5.2e-5*]
		…QSEVQ^▽^LV^▽^ES^▽^GG^▽^GLVQPGGSLR	0.09 [*5.2e-5*]
Herceptin—LC	L1	…RC ^▼^DIQMTQSPSSLSASVGDR	99.18 [*8.1e-4*]
		…RCDIQMTQ^▽^SPSSLSASVGDR	0.71 [7.*2e-4*]
		…RCDIQMTQSP^▽^SSLSASVGDR	0.11 [*2.6e-4*]
Humira—HC	H7	…QC ^▼^EVQLVESGGGLVQPGR	100 [*nil*]
Humira—LC	L1	…RC ^▼^DIQMTQSPSSLSASVGDR	100 [*nil*]
Rituxan—HC	H7	…LS ^▼^QVQLQQPGAELVKPGASVK	99.62 [*5.0e-4*]
		…LSQVQL^▽^QQ^▽^PGAELVKPGASVK	0.38 [*5.0e-4*]
Rituxan—LC	L2	…MA ^▼^QIVLSQSPAILSASPGEK	99.81 [*6.9e-4*]
		…MAQ^▽^I^▽^VLSQSPAI^▽^LSASPGEK	0.19 [*6.9e-4*]
Remicade—HC	H7	…QC ^▼^EVK^↓^LEESGGGLVQPGGSMK	*100.00 [*nil*]
Remicade—LC	L2	…RC ^▼^DILLTQSPAILSVSPGER	100 [*nil*]

Light chain (LC); heavy chain (HC); signal peptide processing site: actual N-terminal (▼), erroneous N-terminal (▽); tryptic site (⬇). Signal peptide sequences are depicted in smaller font size.

Erroneously processed N-terminal peptides >0.5% are presented individually, while those <0.5% are grouped together where applicable.

*Estimation of proportion of Remicade-HC N-terminal peptides may not be accurate as it relied on the quantification of peptides with one missed trypsin cleavage.

## Discussion

As the signal peptide of a nascent protein emerges from the ribosome, it binds the SRP which delivers the SRP-RNC complex to the SR on the ER membrane. The function of the SRP is conserved in bacteria, yeast, plants and mammals [25]. In mammalian cells, the SPR contains one RNA (7S RNA) and six protein molecules [26]. The SRP54 protein and the 7S RNA form the core structure of the SRP complex. The SRP54 protein is universally conserved from *E*. *coli* to human and it contains three domains, the N-domain, the G-domain and the M-domain. The N-terminal helix of the M-domain in human SRP54 and its bacterial homologue, Ffh, extends away from the core structure and forms a large hydrophobic groove. It is this hydrophobic groove that interacts with the hydrophobic H-domain of the signal peptides that commonly contain various numbers of hydrophobic residues [27, 28]. Theoretically, different signal peptides can have different affinities towards the hydrophobic groove of the SRP54 protein.

Several studies have shown that protein production can be enhanced through the use of alternative signal peptides [12–17]. Our work is a more systematic analysis compared to previous publications for identifying the best signal peptides for recombinant antibody production in CHO-K1 cells. We focused on the impact of different antibody signal peptides on the production of 5 top-selling antibody therapeutics. Our strategy for selecting optimal signal peptides involved the generation of a database of known antibody signal peptides of human Ig HCs and kappa LCs from complete cDNA sequences that were available from the public database. Due to the fact that majority of antibody drugs are now either humanized or fully human antibodies, only signal peptides of human origin were collected in our database. Signal peptides of mouse origin will be included in future studies.

Based on sequence similarities, eight HC and two LC signal peptides were fused to each of 5 antibodies for secretion efficiency analysis. Our results showed that some antibodies can tolerate different signal peptides whereas others are more restricted. These results might suggest that part of the variable regions can also affect secretion. For some antibodies (Herceptin, Avastin and Humira), the antibody productivity was higher when L1 LC was utilized, whereas for others (Rituxan and Remicade) L2 LC resulted in higher productivity of the antibody, suggesting that the LC signal peptide also affects the overall productivity of the antibody. Our optimized signal peptides for Rituxan were found to be twice as efficient as its native mouse signal peptides which are available in public database. This result further demonstrates the importance of signal peptide optimization for the production of recombinant antibodies. Our data have clearly shown that when two signal peptides (e.g. H5 and H7) are linked to different antibodies (e.g. Herceptin and Avastin), the impacts of the signal peptides on production of the antibodies can be very different (as shown in Fig. 1: H5-Herceptin >> H5-Avastin, but H7-Avastin >> H7-Herceptin). These observations suggest that, in addition to the signal peptide, a portion of the variable region of the antibody also affects the secretion efficiency. The SRP may interact with both the signal peptide and part of the variable region that will not be cleaved. The findings of this work suggest that signal peptides need to be optimized for all monoclonal antibodies. We believe that similar results obtained in our transient expression experiments are predictable for stably transfected pools. With optimized signal peptides, the expression levels of the 5 antibodies observed in our transient expression experiments follow the order of Herceptin > Rituxan > Avastin > Humira > Remicade (Fig. 2). The same order with respect to expression levels was also observed when stably transfected pools were used to produce these antibodies.

It has been suggested that signal peptides consist of 3 domains, the N-domain, the H-domain and the C-domain [20, 21]. Most of Ig HC signal peptides contain 19 amino acids. The N-domain consists of N-terminal 6 amino acids, followed by the H-domain that contains the hydrophobic core of 8 amino acids. The C-terminal domain contains 5-amino acids that end either with a serine or a cysteine residue. It has been postulated that different signal peptides show varying affinities for the SRP which subsequently determines the efficiency with which a nascent polypeptide chain enters the secretory pathway [14]. An interesting result from our study showed that signal peptide H7 represented the best signal peptide for the HC of majority of the antibodies tested. Substitutions of a signal amino acid in the N-domain (L5W) and the H-domain (V8L and L13A) of the optimal signal peptide (H7) dramatically reduced the productivity of Avastin, suggesting that hydrophobic core of the signal peptide plays a critical role in interacting with the SRP and the translocon [4, 7, 29, 30]. In addition to enhancing secretion efficiency we also showed that the optimized heavy and light chain signal peptides for the 5 antibodies were removed efficiently and accurately by the SPP, resulting in highly homogeneous antibody products.

In summary, we have optimized signal peptide pairs for each of the 5 top-selling antibody drugs. This information is crucial for producing these antibodies as biosimilar drugs. More importantly, this platform technology may also be used to identify the best signal peptides for producing new antibody drugs in the future.

## Supporting Information

S1 Fig Phylogenetic trees of signal peptides from human Ig heavy chains and kappa light chains. A database of signal peptides from 172 human Ig heavy chains and 62 human kappa light chains was generated. The signal peptides were then aligned based on sequence similarity.
(TIF)

S2 Fig Impact of signal peptides on the production of 5 top-selling antibody therapeutics in CHO-K1 cells. Each antibody heavy chain (HC) was fused to 8 different signal peptides (H1 to H8) and each light chain (LC) was fused to 2 signal peptides (L1 and L2). To express each antibody, 8 HC constructs were paired with one LC construct, either L1 or L2. Each graph shows the titers of the same antibody in the conditioned media as determined by ELISA. The Left panels show the results when HCs were paired with L1 LC, while the right panels show the results when HCs were paired with L2 LC. The black bars and the open bars show the titers of the same antibody pair in duplicate experiments. The grey bars (GFP) show the mean fluorescence intensity of GFP which was measured to normalize the transfection efficiency.
(TIF)

S1 Table Detailed information of 172 Ig heavy chain signal peptides and 62 kappa light chain signal peptides listed in the same order as shown in S1 Fig. (DOCX)

S2 Table Proportion of N-terminal peptide(s) quantified by triplicate analyses using mass spectrometry. (DOCX)

## References

[1] Walsh G (2014) Biopharmaceutical benchmarks 2014. Nat Biotechnol 32: 992–1000. 10.1038/nbt.3040 25299917

[2] Nelson AL, Dhimolea E, Reichert JM (2010) Development trends for human monoclonal antibody therapeutics. Nat Rev Drug Discov 9: 767–774. 10.1038/nrd3229 20811384

[3] De Jesus M, Wurm FM (2011) Manufacturing recombinant proteins in kg-ton quantities using animal cells in bioreactors. Eur J Pharm Biopharm 78: 184–188. 10.1016/j.ejpb.2011.01.005 21256214

[4] Egea PF, Stroud RM, Walter P (2005) Targeting proteins to membranes: structure of the signal recognition particle. Curr Opin Struct Biol 15: 213–220. 15837181 10.1016/j.sbi.2005.03.007

[5] Nagai K, Oubridge C, Kuglstatter A, Menichelli E, Isel C, et al (2003) Structure, function and evolution of the signal recognition particle. Embo J 22: 3479–3485. 12853463 10.1093/emboj/cdg337 PMC165607

[6] Wild K, Halic M, Sinning I, Beckmann R (2004) SRP meets the ribosome. Nat Struct Mol Biol 11: 1049–1053. 15523481 10.1038/nsmb853

[7] Hegde RS, Bernstein HD (2006) The surprising complexity of signal sequences. Trends Biochem Sci 31: 563–571. 16919958 10.1016/j.tibs.2006.08.004

[8] Saraogi I, Shan SO (2011) Molecular mechanism of co-translational protein targeting by the signal recognition particle. Traffic 12: 535–542. 10.1111/j.1600-0854.2011.01171.x 21291501 PMC3077218

[9] Johnson AE, van Waes MA (1999) The translocon: a dynamic gateway at the ER membrane. Annu Rev Cell Dev Biol 15: 799–842. 10611978 10.1146/annurev.cellbio.15.1.799

[10] Fluhrer R, Steiner H, Haass C (2009) Intramembrane proteolysis by signal peptide peptidases: a comparative discussion of GXGD-type aspartyl proteases. J Biol Chem 284: 13975–13979. 10.1074/jbc.R800040200 19189970 PMC2682845

[11] Liu J, O’Kane DJ, Escher A (1997) Secretion of functional Renilla reniformis luciferase by mammalian cells. Gene 203: 141–148. 9426244 10.1016/s0378-1119(97)00505-2

[12] Knappskog S, Ravneberg H, Gjerdrum C, Trosse C, Stern B, et al (2007) The level of synthesis and secretion of Gaussia princeps luciferase in transfected CHO cells is heavily dependent on the choice of signal peptide. J Biotechnol 128: 705–715. 17316861 10.1016/j.jbiotec.2006.11.026

[13] Kober L, Zehe C, Bode J (2013) Optimized signal peptides for the development of high expressing CHO cell lines. Biotechnol Bioeng 110: 1164–1173. 10.1002/bit.24776 23124363

[14] Zhang L, Leng Q, Mixson AJ (2005) Alteration in the IL-2 signal peptide affects secretion of proteins in vitro and in vivo. J Gene Med 7: 354–365. 15619290 10.1002/jgm.677

[15] Reisinger H, Steinfellner W, Stern B, Katinger H, Kunert R (2008) The absence of effect of gene copy number and mRNA level on the amount of mAb secretion from mammalian cells. Appl Microbiol Biotechnol 81: 701–710. 10.1007/s00253-008-1701-1 18810429

[16] Futatsumori-Sugai M, Tsumoto K (2010) Signal peptide design for improving recombinant protein secretion in the baculovirus expression vector system. Biochem Biophys Res Commun 391: 931–935. 10.1016/j.bbrc.2009.11.167 19962965

[17] Klatt S, Konthur Z (2012) Secretory signal peptide modification for optimized antibody-fragment expression-secretion in Leishmania tarentolae. Microb Cell Fact 11: 97 10.1186/1475-2859-11-97 22830363 PMC3416730

[18] Yang Y, Ho SC, Yap MG (2009) Mutated polyadenylation signals for controlling expression levels of multiple genes in mammalian cells. Biotechnol Bioeng 102: 1152–1160. 10.1002/bit.22152 18973284

[19] Ho SC, Koh EY, van Beers M, Mueller M, Wan C, et al (2013) Control of IgG LC:HC ratio in stably transfected CHO cells and study of the impact on expression, aggregation, glycosylation and conformational stability. J Biotechnol 165: 157–166. 10.1016/j.jbiotec.2013.03.019 23583871

[20] von Heijne G (1985) Signal sequences. The limits of variation. J Mol Biol 184: 99–105. 4032478 10.1016/0022-2836(85)90046-4

[21] Nicchitta CV (2002) Signal sequence function in the mammalian endoplasmic reticulum: A biological perspective. Current topics in membrane 52: 483–499.

[22] Kotia RB, Raghani AR (2010) Analysis of monoclonal antibody product heterogeneity resulting from alternate cleavage sites of signal peptide. Anal Biochem 399: 190–195. 10.1016/j.ab.2010.01.008 20074542

[23] Ying H, Liu H (2007) Identification of an alternative signal peptide cleavage site of mouse monoclonal antibodies by mass spectrometry. Immunol Lett 111: 66–68. 17583358 10.1016/j.imlet.2007.05.002

[24] Yu XC, Borisov OV, Alvarez M, Michels DA, Wang YJ, et al (2009) Identification of codon-specific serine to asparagine mistranslation in recombinant monoclonal antibodies by high-resolution mass spectrometry. Anal Chem 81: 9282–9290. 10.1021/ac901541h 19852494

[25] Pool MR (2005) Signal recognition particles in chloroplasts, bacteria, yeast and mammals (review). Mol Membr Biol 22: 3–15. 16092520 10.1080/09687860400026348

[26] Walter P, Blobel G (1982) Signal recognition particle contains a 7S RNA essential for protein translocation across the endoplasmic reticulum. Nature 299: 691–698. 6181418 10.1038/299691a0

[27] Clemons WM Jr, Gowda K, Black SD, Zwieb C, Ramakrishnan V (1999) Crystal structure of the conserved subdomain of human protein SRP54M at 2.1 A resolution: evidence for the mechanism of signal peptide binding. J Mol Biol 292: 697–705. 10497032 10.1006/jmbi.1999.3090

[28] Janda CY, Li J, Oubridge C, Hernandez H, Robinson CV, et al (2010) Recognition of a signal peptide by the signal recognition particle. Nature 465: 507–510. 10.1038/nature08870 20364120 PMC2897128

[29] Halic M, Beckmann R (2005) The signal recognition particle and its interactions during protein targeting. Curr Opin Struct Biol 15: 116–125. 15718142 10.1016/j.sbi.2005.01.013

[30] Doudna JA, Batey RT (2004) Structural insights into the signal recognition particle. Annu Rev Biochem 73: 539–557. 15189152 10.1146/annurev.biochem.73.011303.074048

