# Supplementary figures and images for: Optimization of Heavy Chain and Light Chain Signal Peptides for High Level Expression of Therapeutic Antibodies in CHO Cells
Source: PLoS One. 2015 Feb 23;10(2):e0116878. doi: 10.1371/journal.pone.0116878 (PMC4338144; doi:10.1371/journal.pone.0116878)

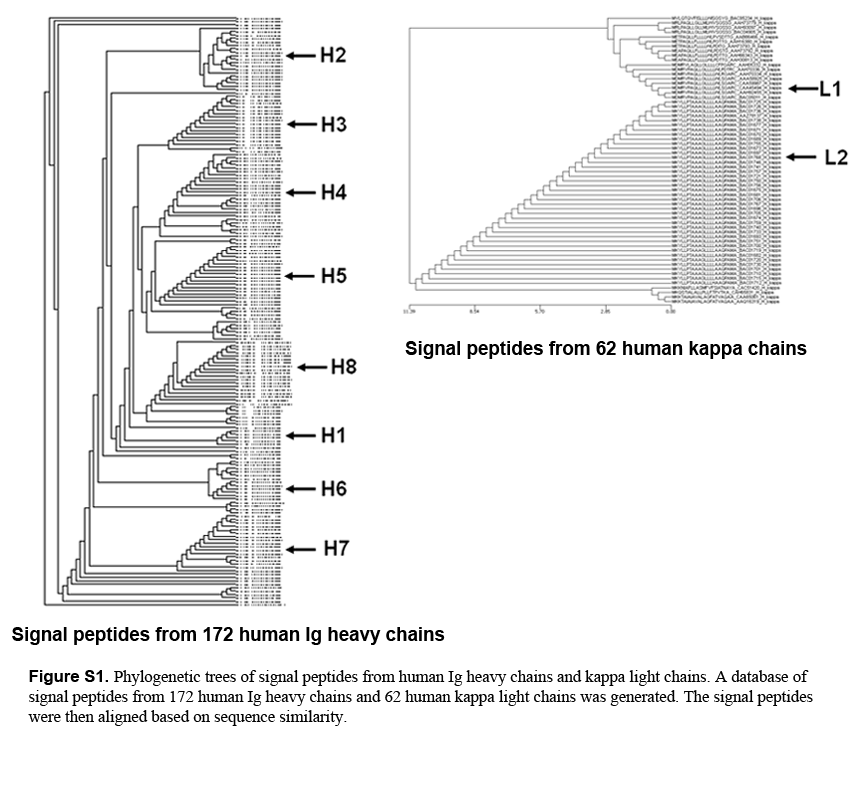

Supplement: S1 Fig — A database of signal peptides from 172 human Ig heavy chains and 62 human kappa light chains was generated. The signal peptides were then aligned based on sequence similarity. (TIF) [file pone.0116878.s001.tif]

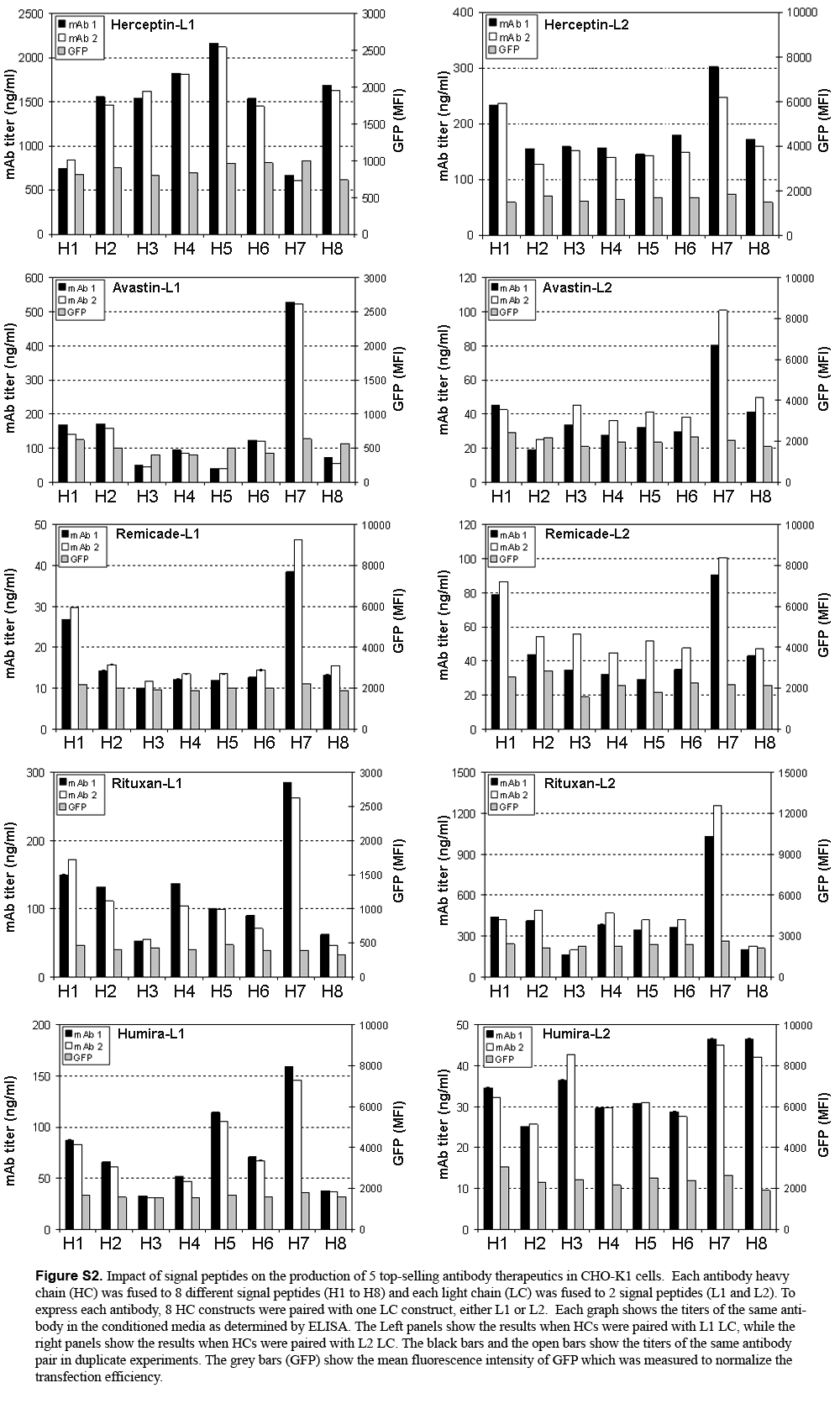

Supplement: S2 Fig — Each antibody heavy chain (HC) was fused to 8 different signal peptides (H1 to H8) and each light chain (LC) was fused to 2 signal peptides (L1 and L2). To express each antibody, 8 HC constructs were paired with one LC construct, either L1 or L2. Each graph shows the titers of the same antibody in the conditioned media as determined by ELISA. The Left panels show the results when HCs were paired with L1 LC, while the right panels show the results when HCs were paired with L2 LC. The black bars and the open bars show the titers of the same antibody pair in duplicate experiments. The grey bars (GFP) show the mean fluorescence intensity of GFP which was measured to normalize the transfection efficiency. (TIF) [file pone.0116878.s002.tif]
