# Supplementary material for: Optimization of Heavy Chain and Light Chain Signal Peptides for High Level Expression of Therapeutic Antibodies in CHO Cells
Source: PLoS One. 2015 Feb 23;10(2):e0116878. doi: 10.1371/journal.pone.0116878 (PMC4338144; doi:10.1371/journal.pone.0116878)
Supplement: S1 Table — (DOCX) [file pone.0116878.s003.docx]

**Table S1**. Detailed information of 172 Ig heavy chain signal peptides and 62 kappa light chain signal peptides listed in the same order as shown in Figure S1

| **Heavy chain** | **Amino acid sequence** | **GenBank accession number** | **Isotype** |
| --- | --- | --- | --- |
|  | MEFGLRWVFLVAILKDVQC | CAA75030 | IgG1 |
|  | MEFGLSWVFLVAILKGVQC | BAC04996 | IgG2 |
|  | MEFGLSWVFLVAILKGVQC | BAC85172 | IgG1 |
|  | MELGLSWVFLVAILKGVQC | BAC87456 | IgA1 |
|  | MELGLRWVFLVAFLEGVQC | BAB55072 | IgA1 |
|  | MELGLRWVFLVTFFWGVQC | BAC85355 | IgA1 |
|  | MELGLRWVLLVAILEGVHC | BAC87542 | IgD |
|  | MELGLRWVFLVALLEGVHC | BAC86968 | IgG1 |
|  | MELGLRWVFLIATLAGARC | BAC05016 | IgG1 |
| **H2** | MELGLRWVFLVAILEGVQC | AAS01769 | IgM |
|  | MELGLRWVFLVAILEGVQC | CAE45931 | IgG2 |
|  | MELGLYWVFLVAILEGVQC | BAC87558 | IgA1 |
|  | MDLGLYWVFLVAILEGVEC | BAC86537 | IgG3 |
|  | MELGLCWVFLVAILEGVPC | CAE45921 | IgA2 |
|  | MELGLCWVFLVAILEGVQC | BAG62903 | IgG3 |
|  | MELGLNWVLLVAILEGVQC | AAH62335 | IgG2 |
|  | MELGLSWVFLVAILEGVHC | BAC85740 | IgA2 |
|  | MELGLSWVFLVAILEGVQC | CAE45898 | IgA2 |
|  | MELGLSWVFLVAILEGVQC | BAC85395 | IgG2 |
|  | MELGLSWVFLVAILEGVQC | AAH64496 | IgG1 |
|  | MELGLSWVFLVAILEGVQC | AAH41037 | IgG1 |
|  | MELGLSWVFLVVILEGVQC | AAH53984 | IgG1 |
|  | MESGLTWLFLVAILKGVHC | BAC85363 | IgA1 |
| **H3** | MKHLWFFLLLVAAPRWVLS | AAH16369 | IgA1 |
|  | MKHLWFFLLLVAPPRWVLS | BAC85432 | IgA1 |
|  | MKHLWFFLLLVATPRWVLS | AAH73767 | IgM |
|  | MKHLWFFLLLVAAPRWVLS | BAC85359 | IgA1 |
|  | MKHLWFFLLLVAAPRWVLS | AAH73765 | IgA2 |
|  | MKHLWFFLLLVAAPRWVLS | BAC86044 | IgA2 |
|  | MKHLWFFLLLVAAPRWVLS | AAH02963 | IgM |
|  | MKHLWFFLLLVAAPRWVLS | BAC87432 | IgG2 |
|  | MKHLWFFLLLVAAPRWVLS | BAC85436 | IgG1 |
|  | MKHLWFFLLLVAAPRWVLS | AAH73773 | IgG1 |
|  | MKHLWFFLLLVAAPRWVLS | AAH73766 | IgG1 |
|  | MKHLWFFLLLVAAPRWVLS | AAH75842 | IgG1 |
|  | MKHLWFFLLLVAAPRWVLS | BAC85387 | IgG1 |
|  | MRHLWFFLLLVAAPRWVLS | BAC87510 | IgD |
|  | MKHLWFFFLLVAAPRSVLS | BAC85198 | IgA1 |
|  | MSVSFLIFLPVLGLPWGVLS | AAS01770 | IgM |
|  | MGHPWFFLLLVTAPRWVLS | BAC05021 | IgG1 |
| **H4** | MDWTWRILFLVAAATGAHS | AAH87841 | IgA1 |
|  | MDWTWRILFLVAAATDAYS | AAH09851 | IgM |
|  | MDWTWRILFLVAAATSAHS | BAG62882 | IgG3 |
|  | MDWTWRILFLVAAATSAHS | AAH37361 | IgG1 |
|  | MDWTWRILFLVAAATEAHS | BAC85175 | IgG1 |
|  | MDWTWRILFLVTAATGAHS | CAE45841 | IgG3 |
|  | MDWTWRILFLVAAATGAHS | CAE45917 | IgA1 |
|  | MDWTWRILFLVAAATGAHS | BAC85440 | IgE |
|  | MDWTWRILFLVAAATGAHS | AAI11020 | IgG4 |
|  | MDWTWRILFLVAAATGAHS | AAH80557 | IgG1 |
|  | MDWTWRILFLVAAATGAHS | AAH75840 | IgG1 |
|  | MDWTWRILFLVAAATGAHS | BAC85235 | IgG1 |
|  | MDWTWRLLFLVAAVTSAHS | AAL36987 | IgA1 |
|  | MDWTWSILFLVAAATGAHS | BAG62928 | IgG1 |
|  | MDWTWSILFLVTAATGAHS | BAG62876 | IgG1 |
|  | MDWTWSILFLVAGASGAHS | AAH73789 | IgG1 |
|  | MDWTWSILFLVAAATGARP | AAH51328 | IgG1 |
|  | MGWTWSILFLVAATTGAPS | CAE45780 | IgA1 |
|  | MDWTWSILFLVAAATGAQS | AAH05951 | IgA1 |
|  | MDWAWRILFLVAAATGVHS | BAC86585 | IgA1 |
|  | MDCTWRILLLVAVATGTHA | AAH89421 | IgG3 |
|  | MDCTWRILLLVAAATGTHA | AAH90939 | IgG1 |
|  | MDWTWRILFLAAAATGVQS | AAH65733 | IgA2 |
|  | MDWTWTILFLVAGATGVKS | BAC86514 | IgG1 |
|  | MDWTWSILFLVAAATGVHS | CAE45779 | IgA2 |
|  | MDWTWRFLFVVAAVTGVQS | BAB71633 | IgA1 |
|  | MDWTWRFLFVVAAVTGVQS | AAH67091 | IgG1 |
|  | MDWTWILFLVAAATRVHS | AAB59424 | IgE |
|  | MDWTWRFLLVVAAATGVPS | AAH75846 | IgG1 |
|  | MDWTWRFLIVVAAATGVQS | BAC85173 | IgG1 |
|  | MDWTWRFLFVVAAATSVQS | AAH25314 | IgG1 |
| **H5** | MDWTWRFLFVVAAATGVQS | CAA34971 | IgM |
|  | MDWTWRFLFVVAAATGVQS | BAC86457 | IgG3 |
|  | MDWTWRFLFVVAAATGVQS | BAC05013 | IgG1 |
|  | MDWTWRFLFVVAAATGVQS | BAC85429 | IgG1 |
|  | MDWTWRFLFVVAAATGVQS | AAH62336 | IgG1 |
|  | MDWTWRFLFVVAAATGVQS | AAA02914 | IgG1 |
|  | MDWTWRFLFVVAAATGVQS | AAH69016 | IgG1 |
|  | MDWTWRFLFVVAAATGVQS | BAC05017 | IgG1 |
|  | MDWTWRFLFVVAAATGVQS | BAC87418 | IgG1 |
|  | MDWTWRFLFVVAAATGVQS | AAO17821 | IgG1 |
|  | MDWTWRFLFVVAAATGVQS | AAH16381 | IgG1 |
|  | MDWTWRFLFVVAAGTGVQS | AAH26038 | IgG1 |
|  | MDWTWRFLFVVAASTGVQS | BAC05020 | IgG1 |
|  | MDWTWRVLFVVAASTGVQS | BAC85529 | IgG1 |
|  | MDRTWRLLFVVAAATGVQS | BAC85401 | IgG1 |
|  | MDWTWRFLFVVAAAAGVQS | BAC85697 | IgG1 |
|  | MGWTWRFLFVVAAAAGVQS | BAC05018 | IgG1 |
|  | MDWTWTFLFVVAAATGVQS | BAC85199 | IgG1 |
|  | MDWTWRVFCLLAVAPGVQS | BAC86094 | IgA1 |
|  | MDWTWRVFCLLAVAPGADS | CAE45829 | IgA2 |
|  | MDWTWRVFCLLAVAPGANS | BAC05012 | IgG1 |
|  | MDWTWRVFCLLAVAPGAHS | AAH19337 | IgG1 |
|  | MDWTWRVFCLLAVISGGQS | BAC05014 | IgG1 |
|  | MDWTWRFLFVVAVAIGVQS | BAC85483 | IgG2 |
|  | MDLMCKKMKHLWFFLLLVAAPRWVLS | BAC85190 | IgA1 |
| **H8** | MDLLHKNMKHLWFFLLLVAAPRWVLS | BAC87192 | IgA1 |
|  | MGLLHKNMKHLWFFLLLVAAPRWVLS | AAH63384 | IgD |
|  | MDLLHKNMKHLWFFLLLVAAPRWVLS | BAC85361 | IgA1 |
|  | MDLLHKNMKHLWFFLLLVAAPRWVLS | AAH19235 | IgM |
|  | MDLLHKNMKHLWFFLLLVAAPRWVLS | AAH17356 | IgM |
|  | MDLLHKNMKHLWFFLLLVAAPRWVLS | AAH11857 | IgM |
|  | MDLLHKNMKHLWFFLLLVAAPRWVLS | BAG62978 | IgG3 |
|  | MDLLHKNMKHLWFFLLLVAAPRWGLS | BAC87548 | IgD |
|  | MDVMCKKMKHLWFFLLLVAAPRWVLA | BAB71560 | IgG1 |
|  | MDLKCKKMKRLWLFLLLVAAPRWVLS | BAC85394 | IgG1 |
|  | MDLLCKNMKHLWFFLLLVAAPRWVLS | AAH25985 | IgG4 |
|  | MDLLCKNMKHLWFFLLLVAAPRWVLS | BAG64279 | IgG1 |
|  | MDLLCKKMKHLWFFLLLVAAPRWVLS | BAC85174 | IgG1 |
|  | MDLMCKKMKHLWFFLLLVAAPRWVLS | BAC85371 | IgA1 |
|  | MELMCKKMKHLWFFLLLVAAPRWVLS | CAE45900 | IgG4 |
|  | MDLMCKKMKHLWFFLLLVAAPGWVLS | BAC86532 | IgA2 |
|  | MCKTMKQLWFFLLLVAAPRWVLS | BAC85393 | IgG2 |
|  | MAKTNLFLFLIFSLLLSLSSAAQPAMA | AAV67804 | IgG1 |
|  | MDTLCSTLLLLTIPSWVLS | AAH92449 | IgA1 |
|  | MDTLCSTLLLLTIPSWVLS | AAO17822 | IgG3 |
|  | MDTLCSTLLLLTIPSWVLS | BAG62883 | IgG1 |
|  | MGSTAILALLLAVLQGVCA | AAH14258 | IgG1 |
|  | MGSTAILALLLAVLQGVCA | AAH89417 | IgG1 |
|  | MGSTAILALLLAVLQGVCA | AAH78670 | IgG1 |
|  | MELSLSWFFLLTIIQGVQC | BAC85641 | IgA1 |
| **H1** | MELGLSWIFLLAILKGVQC | BAC87503 | IgA1 |
|  | MELGLSWIFLLAILKGVQC | AAH21276 | IgD |
|  | MELGLSWIFLLAILKGVQC | AAH06402 | IgG1 |
|  | MDLGLSWIFLLTILKGVQC | BAC85171 | IgG1 |
|  | MELGLTWIFLLAILKGVQC | CAE45781 | IgG1 |
|  | MELGLSWIFLVAILKGVQC | BAC87538 | IgD |
|  | MDLGLSWLFLVALLKGVQC | BAC05203 | IgG1 |
|  | MEFGLSCVFLVAIFKGVHC | AAH73782 | IgG1 |
|  | MEFGLSCLFLVAILKGVRC | AAH78671 | IgG1 |
|  | MEFGLSWIFLVVIIKGVQC | BAC86210 | IgA1 |
|  | MEFGLSWIFLVVILKGVQC | BAC85388 | IgG1 |
|  | MEFGLSWIFLATILKGVQC | BAC85350 | IgG1 |
|  | MEFGLSWIFLAAILKGVQC | BAF82062 | IgG1 |
|  | MEFGLSWIFLAAILKGVQG | AAH65820 | IgG1 |
|  | MKFGLSWIFLPAILKGVQC | AAH14667 | IgG1 |
| **H6** | MEFGLSWLFLVAILKGVQC | AAH15760 | IgM |
|  | MEFGLSWLFLVAILKGVQC | BAG62929 | IgG3 |
|  | MEFGLSWLFLVAILKGVQC | BAC04926 | IgG1 |
|  | MEFGLSWLFLVAILKGVQC | AAO17823 | IgG1 |
|  | MEFGLSWLLLVAILKGVQC | AAH69020 | IgG1 |
|  | MEFGLSWLFLVTILKGVQC | BAC85232 | IgG1 |
|  | MEFGLSWVFLVAIIKGVQCQV | AAH73758 | IgM |
|  | MEFGLSWVFLVAIIKGVQC | BAC85444 | IgG1 |
|  | MEFGLSWVFLVAVIKGVQC | BAG62938 | IgG3 |
|  | MEFGLTWVFLVAVIKGVHC | BAC85373 | IgG1 |
|  | MQFGLSWVFLVALLRGVQC | BAC11114 | IgA1 |
|  | MDFGLAWVFLVALLRGVQC | BAC87529 | IgD |
|  | MEFGLNWVLLVALLRGVQC | BAC05022 | IgG1 |
|  | MEFGLSWVFLVALLRGVQC | BAC87554 | IgD |
|  | MEFGLSWVFLVALLRGVQC | CAE45778 | IgA1 |
|  | MEFGLSWVFLVALLRGVEC | BAC87380 | IgG1 |
|  | MEFGLSWVFLVALLRGVQC | AAH20240 | IgM |
|  | MEFGLSWVFLVALLRGVQC | CAH18705 | IgG2 |
|  | MEFGLSWVFLVALLRGVQC | BAD08204 | IgG1 |
|  | MEFGLSWVFLVALLRGVQC | AAW82028 | IgG1 |
|  | MEFGLSWVFLVALLRGVQC | BAC04208 | IgG1 |
| **H7** | MEFGLSWVFLVALFRGVQC | BAC87457 | IgG4 |
|  | MESGLSWVFLVALLRGVQC | BAC86513 | IgA1 |
|  | MELGLSWVFLVSLLAGVQC | BAC85366 | IgA1 |
|  | MELGLSWIFLVALLRGVQC | CAE45773 | IgG1 |
|  | MEFGLSWVLLVVFLQGVQC | AAH33178 | IgG3 |
|  | MEFGLSWVFLVGILKGVQC | BAC85349 | IgA1 |
|  | MEFGLSWVYLVAILKGVQC | BAC04226 | IgG1 |
|  | MEFGLSWVFLVAILKGVQC | AAH32249 | IgA1 |
|  | MEFGLSWVFLVAILKGVQC | AAH90938 | IgG1 |
|  | MEFGLSWVFLVAILKGVQC | BAC86964 | IgG1 |
|  | MEFGLSWVFLVAILKGVQC | AAH73771 | IgA1 |
|  | MEFWLSWVFLVAILKGVQC | AAH72419 | IgG1 |
|  | MEFGLSWVFLVAILKGVQC | AAT49050 | IgG1 |
|  | MEFGLSWVFLVAILKGVQC | AAH92518 | IgG1 |
|  | MEFGLSWVFLVAILKGVQC | BAC86225 | IgG1 |
|  |  |  |  |
|  |  |  |  |
| **Light chain** | **Amino acid sequence** | **GenBank accession number** | **Isotype** |
|  | MVLQTQVFISLLLWISGSYG | BAC85234 | kappa |
|  | MRLPAQLLGLLMLWVSGSSG | AAH73779 | kappa |
|  | MRLPAQLLGLLMLWVSGSSG | AAH93097 | kappa |
|  | MRLPAQLLGLLMLWVSGSSG | BAC04905 | kappa |
|  | METPAQLLFLLLLWLPVSDTTG | AAB86466 | kappa |
|  | METPAQLLFLLLLWLPGTTG | AAH16380 | kappa |
|  | METPAQLLFLLLLWLPDITG | AAH73793 | kappa |
|  | MEAPAQLLFLLLLWLPDSTG | AAH73792 | kappa |
|  | MEAPAQLLFLLLLWLPDTTG | AAH66343 | kappa |
|  | MEAPAQLLFLLLLWLPDTTG | AAH30813 | kappa |
|  | MDMRVLAQLLGLLLLCFPGARC | AAH05332 | kappa |
|  | MDMRVPAQLLGLLLLWLPDTRC | AAH70336 | kappa |
|  | MDMRVPAQLLGLLLLWLRGARC | AAH70334 | kappa |
| **L1** | MDMRVPAQLLGLLLLWLSGARC | AAA58925 | kappa |
|  | MDMRVPAQLLGLLLLWLSGARC | CAA59987 | kappa |
|  | MDMRVPAQLLGLLLLWLSGARC | CAA45494 | kappa |
|  | MDMRVPAQLLGLLLLWLSGARC | AAH92455 | kappa |
|  | MDMRVPAQLLGLLLLWLSGARC | BAC05011 | kappa |
| **L2** | MKYLLPTAAAGLLLLAAQPAMA | BAC01725 | kappa |
|  | MKYLLPTAAAGLLLLAAQPAMA | BAC01726 | kappa |
|  | MKYLLPTAAAGLLLLAAQPAMA | BAC01736 | kappa |
|  | MKYLLPTAAAGLLLLAAQPAMA | AAZ78137 | kappa |
|  | MKYLLPTAAAGLLLLAAQPAMA | BAC01738 | kappa |
|  | MKYLLPTAAAGLLLLAAQPAMA | BAC01677 | kappa |
|  | MKYLLPTAAAGLLLLAAQPAMA | BAC01671 | kappa |
|  | MKYLLPTAAAGLLLLAAQPAMA | BAC01670 | kappa |
|  | MKYLLPTAAAGLLLLAAQPAMA | BAC01689 | kappa |
|  | MKYLLPTAAAGLLLLAAQPAMA | BAC01755 | kappa |
|  | MKYLLPTAAAGLLLLAAQPAMA | BAC01673 | kappa |
|  | MKYLLPTAAAGLLLLAAQPAMA | BAC01697 | kappa |
|  | MKYLLPTAAAGLLLLAAQPAMA | BAC01768 | kappa |
|  | MKYLLPTAAAGLLLLAAQPAMA | BAC01746 | kappa |
|  | MKYLLPTAAAGLLLLAAQPAMA | BAC01741 | kappa |
|  | MKYLLPTAAAGLLLLAAQPAMA | BAC01757 | kappa |
|  | MKYLLPTAAAGLLLLAAQPAMA | BAC01721 | kappa |
|  | MKYLLPTAAAGLLLLAAQPAMA | BAC01750 | kappa |
|  | MKYLLPTAAAGLLLLAAQPAMA | BAC01756 | kappa |
|  | MKYLLPTAAAGLLLLAAQPAMA | BAC01675 | kappa |
|  | MKYLLPTAAAGLLLLAAQPAMA | BAC01681 | kappa |
|  | MKYLLPTAAAGLLLLAAQPAMA | BAC01706 | kappa |
|  | MKYLLPTAAAGLLLLAAQPAMA | BAC01767 | kappa |
|  | MKYLLPTAAAGLLLLAAQPAMA | BAC01696 | kappa |
|  | MKYLLPTAAAGLLLLAAQPAMA | BAC01705 | kappa |
|  | MKYLLPTAAAGLLLLAAQPAMA | BAC01674 | kappa |
|  | MKYLLPTAAAGLLLLAAQPAMA | BAC01759 | kappa |
|  | MKYLLPTAAAGLLLLAAQPAMA | BAC01740 | kappa |
|  | MKYLLPTAAAGLLLLAAQPAMA | BAC01733 | kappa |
|  | MKYLLPTAAAGLLLLAAQPAMA | BAC01693 | kappa |
|  | MKYLLPTAAAGLLLLAAQPAMA | BAC01751 | kappa |
|  | MKYLLPTAAAGLLLLAAQPAMA | BAC01700 | kappa |
|  | MKYLLPTAAAGLLLLAAQPAMA | BAC01719 | kappa |
|  | MKYLLPTAAAGLLLLAAQPAMA | BAC01682 | kappa |
|  | MKYLLPTAAAGLLLLAAQPAMA | BAC01720 | kappa |
|  | MKYLLPTAAAGLLLLAAQPAMA | BAC01730 | kappa |
|  | MKYLLPTAAAGLLLLAAQPAMA | BAC01701 | kappa |
|  | MKYLLPTAAAGLLLLAAQPAMA | BAC01710 | kappa |
|  | MKYLLPTAAAGLLLLAAQPAMA | BAC01715 | kappa |
|  | MKYLLPTAAAGLLLHAAQPAMA | BAC01712 | kappa |
|  | MKKNIAFLLASMFVSIATNAYA | CAC51420 | kappa |
|  | MKQSTIALALLPLLFTPVTKA | CAH65831 | kappa |
|  | MKKTAIAIAVALAGFATVAQAA | CAA65061 | kappa |
|  | MKKTAIAIAVALAGFATVAQAA | AAQ16319 | kappa |
