# Supplementary material for: Optimization of Heavy Chain and Light Chain Signal Peptides for High Level Expression of Therapeutic Antibodies in CHO Cells
Source: PLoS One. 2015 Feb 23;10(2):e0116878. doi: 10.1371/journal.pone.0116878 (PMC4338144; doi:10.1371/journal.pone.0116878)
Supplement: S2 Table — (DOCX) [file pone.0116878.s004.docx]

**Table S2.** **Proportion of N-terminal peptide(s) quantified by triplicate analyses using mass spectrometry.**

| **Antibody** | **Signal peptide** | **N-terminal sequence processing** | **Proportion (%)** | | | **Average (%) [SEM]** | |
| --- | --- | --- | --- | --- | --- | --- | --- |
|  |  |  | **1** | **2** | **3** |  |  |
| Avastin – HC  Avastin – LC | H7  L1 | …QC^⯆^EVQLVESGGGLVQPGGSLR  …QCEVQLV^⯆^ESGGGLVQPGGSLR  …RC^⯆^DIQMTQSPSSLSASVGDR | 99.41  0.59  100.00 | 99.29  0.71  100.00 | 99.43  0.57  100.00 | 99.38  0.62  100.00 | [4.5e-4]  [4.5e-4]  [nil] |
| Herceptin – HC  Herceptin – LC | H5  L1 | …QS^⯆^EVQLVESGGGLVQPGGSLR  …QS EVQ^⯆^LVESGGGLVQPGGSLR  …QS EVQLV^⯆^ESGGGLVQPGGSLR  …QS EVQLVES^⯆^GGGLVQPGGSLR  …QS EVQLVESGG^⯆^GLVQPGGSLR  …RC^⯆^DIQMTQSPSSLSASVGDR  …RC DIQMTQ^⯆^SPSSLSASVGDR  …RC DIQMTQSP^⯆^SSLSASVGDR | 99.91  0.02  0.03  0.03  0.01  99.16  0.68  0.16 | 99.92  0.02  0.02  0.02  0.02  99.34  0.59  0.07 | 99.90  0.02  0.03  0.04  0.01  99.06  0.84  0.10 | 99.91  0.02  0.03  0.03  0.01  99.18  0.71  0.11 | [5.2e-5]  [1.3e-5]  [9.6e-6]  [5.0e-5]  [1.6e-5]  [8.1e-4]  [7.2e-4]  [2.6e-4] |
| Humira – HC  Humira – LC | H7  L1 | …QC^⯆^EVQLVESGGGLVQPGR  …RC^⯆^DIQMTQSPSSLSASVGDR | 100.00  100.00 | 100.00  100.00 | 100.00  100.00 | 100.00  100.00 | [nil]  [nil] |
| Rituxan – HC  Rituxan – LC | H7  L2 | …LS^⯆^QVQLQQPGAELVKPGASVK  …LS QVQL^⯆^QQPGAELVKPGASVK  …LS QVQLQQ^⯆^PGAELVKPGASVK  …MA^⯆^QIVLSQSPAILSASPGEK  …MA Q^⯆^IVLSQSPAILSASPGEK  …MA QI^⯆^VLSQSPAILSASPGEK  …MA QIVLSQSPAI^⯆^LSASPGEK | 99.53  0.38  0.09  99.94  0.03  0.01  0.01 | 99.71  0.25  0.05  99.74  0.02  0.05  0.19 | 9.61  0.27  0.13  99.74  0.11  0.14  0.01 | 99.62  0.30  0.09  99.81  0.05  0.07  0.07 | [5.0e-4]  [4.0e-4]  [2.3e-4]  [6.9e-4]  [2.8e-4]  [3.9e-4]  [5.9e-4] |
| Remicade – HC  Remicade – LC | H7  L2 | …QC^⯆^EVKLEESGGGLVQPGGSMK  …QC EVK^🠗^LEESGGGLVQPGGSMK  …RC^⯆^DILLTQSPAILSVSPGER  …RC^⯆^DILLTQSPAILSVSPGERVSFSCR | 2.13  97.87  100.00  absent | 100.00  absent  92.87  7.13 | 100.00  absent  95.91 4.09 | *100.00  100.00 | [nil]  [nil] |

Light chain (LC); heavy chain (HC); signal peptide processing site: actual N-terminal (⯆); erroneous N-terminal (⯆); tryptic site (🠗). Signal peptide sequences are depicted in smaller font size.
*The presence of a tryptic site near the N-terminal of Remicade-HC required reliance on an N-terminal peptide with one missed cleavage for identification and quantification. As such, estimation of the proportion of N-terminal peptides is likely unreliable as majority of the N-terminal peptides would have been processed at the tryptic site, leading to difficulty in identifying any erroneous N-terminal peptides that, if present, would have been at low concentrations to begin with. Note that Remicade sample-1 was digested under normal conditions as described while sample-2 and -3 were digested under conditions that favoured partial digestion (at pH 7.4, room temperature for 4hr).
